# Pressure relieving support surfaces: a randomised evaluation 2 (PRESSURE 2)

**DOI:** 10.1186/1745-6215-14-S1-P68

**Published:** 2013-11-29

**Authors:** Sarah Brown, Isabelle Smith, Julia Brown, Claire Hulme, Jane Nixon

**Affiliations:** 1 Clinical Trials Research Unit, University of Leeds, Leeds, UK; 2 Leeds Institute of Health Sciences, University of Leeds, Leeds, UK

## 

PRESSURE 2 is a multicentre, randomised, double triangular group sequential, parallel group trial involving 2954 patients, comparing High Specification Foam (HSF) and Alternating Pressure Mattresses (APMs) in high risk acute patients for the prevention of new pressure ulcers (PUs). The primary endpoint is time to developing a new Category ≥2 PU from randomisation to 30 days post trial completion (maximum 90 days).

HSF reduces PU incidence compared to the 'traditional standard' hospital mattresses and HSF is the 'new standard' NHS provision. Based upon expert consensus APMs are routinely used for PU prevention in high risk patients or when HSF has failed, and many clinicians lack equipoise in mattress choice in clinical practice. The PRESSURE 2 trial is an efficient design as it allows for early stopping by demonstrating effectiveness of either mattress or futility of the trial, and therefore evidence will potentially be available earlier to guide clinical practice than in a conventional fixed design trial. The trial will use an early primary endpoint so information to assess early stopping will be available in a timely fashion.

Considerations in the design of the group sequential trial included the criteria for early stopping, the number and spacing of planned interim analyses, and the choice of the spending functions for the stopping boundaries. A further consideration was the methodology of a blinding validation sub-study to evaluate any bias in the reporting (under or over-reporting) of pressure ulcers, as it is not possible to blind patients or the clinical team conducting the assessments.

